# Survivability of patients admitted for stroke in a primary stroke center, Penang, Malaysia: a retrospective 5-year study

**DOI:** 10.1186/s40360-023-00669-8

**Published:** 2023-05-02

**Authors:** Monica Danial, Nurul Shahira Izwani Mohdradzi, Amer Hayat Khan, Alan Swee Hock Ch’ng, Looi Irene

**Affiliations:** 1 Clinical Research Centre (CRC) Hospital Seberang Jaya, Institute for Clinical Research, Ministry of Health Malaysia (MOH), Penang, Malaysia; 2 Discipline of Social and Administrative Pharmacy, School of Pharmaceutical Sciences, Universiti Sains Malaysia, Penang, Malaysia; 3 Medical Department, Hospital Seberang Jaya, Ministry of Health Malaysia (MOH), Penang, Malaysia

**Keywords:** Stroke category, Stroke episode, Survival rate, Major drug classes, Hemorrhagic stroke

## Abstract

**Background:**

Stroke is one of the most common noncommunicable diseases, with significant public health implications both globally and in Malaysia. The aim of this study was to evaluate post-stroke survivability as well as the major drug classes prescribed for hospitalized stroke patients.

**Methods:**

A 5-year retrospective study was carried out on the survival of stroke patients admitted to Hospital Seberang Jaya, a main stroke center in the state of Penang, Malaysia. Patients admitted for stroke were first identified using the local stroke registry database, and their medical records were then accessed for data collection, which included demographic information, comorbid conditions, and medications prescribed during admission.

**Results:**

The Kaplan-Meier overall survivability analysis performed indicated 50.5% survival for the duration of 10 days (p < 0.001) post-stroke. Ten-day survivability differences (p < 0.05) were observed for the categories of type of stroke (ischemic stroke (60.9%) and hemorrhagic stroke (14.1%)); stroke episodes (first (61.1%) and recurrent (39.6%)); anti-platelets (prescribed (46.2%) and not prescribed (41.5%)); statins (prescribed (68.7%) and not prescribed (28.1%)); anti-hypertensive (prescribed (65.4%) and not prescribed (45.9%)); and anti-infectives (prescribed (42.5%) and not prescribed (59.6%)) respectively. Higher risks of mortality were observed among patients with hemorrhagic stroke (HR: 10.61, p = 0.004); with 3 or more comorbidities (HR:6.60, p = 0.020); and not prescribed with statins and anti-diabetic. Patients prescribed anti-infectives, on the other hand, had a higher risk of mortality when compared to patients who did not receive anti-infectives (HR: 13.10, p = 0.019). The major drug classes prescribed for stroke patients were antiplatelet drugs (86.7%), statins (84.4%), and protein pump inhibitors (75.6%).

**Conclusion:**

The findings of the study are intended to encourage more non-stroke hospitals in Malaysia to increase their efforts in treating stroke patients, as early treatment can help reduce the severity of the stroke. With the incorporation of evidence-based data, this study also contributes to local data for comparison and improves the implementation of regularly prescribed stroke medication.

## Background

Stroke continues to be the world’s second-leading cause of death and the third-leading cause of death and disability. Progressively from 1990 up to 2019, the burden of stroke increased substantially in terms of incidence, prevalence, and disability-adjusted life-years lost (DALYs)[1]. Various studies have been conducted from different regions of the world, including the World Health Organization monitoring trends and determinants in cardiovascular disease (WHO MONICA project), European countries, the United States, and Australia that reports on the short, medium, and long-term mortality after stroke and the factors that influence it [2–5]. Compared to many other Southeast Asian countries, Malaysia has low age- and sex-standardized stroke mortality and DALYs [6].

Moreover, in the past two decades, ischemic and hemorrhagic stroke incidence and mortality have declined in high-income countries. In contrast, the incidence of hemorrhagic and ischemic stroke increased by 22% and 6% in low-income and middle-income countries, respectively [7]. In Malaysia, the most common type of stroke is ischemic stroke, which accounts for 79.4% of all stroke cases, and followed by hemorrhagic strokes (18.2%) [8].

The most common sites of hemorrhage are the basal ganglia, cerebral lobes, thalamus, pons and brain stem, and cerebellum. Hematomas destroy neurons and glia. This leads to anemia, neurotransmitter release, mitochondrial dysfunction, and cell swelling. Thrombin activates microglia, causing inflammation and edema. Primary injury is due to hematoma compression of brain tissue and increased intracranial pressure, while secondary injury is caused by inflammation, blood-brain barrier disruption, edema, reactive oxygen species, and overproduction of free radicals such as glutamate. It induces excitotoxicity and releases hemoglobin and iron from the clot. A hematoma usually expands within 3 to 12 h. Hematoma expansion occurs within 3 h in one-third of cases [9–11].

Ischemic stroke is caused by either a thrombosis or an embolism that causes a decrease in blood flow to the brain. In thrombotic events, blood flow through the blood vessels to the brain is impeded by the dysfunction of the blood vessels themselves, usually as a result of atherosclerosis, arterial dissection, fibromuscular dysplasia, or inflammatory conditions[12]. In an embolism, debris from elsewhere in the body blocks blood flow through the affected vessel. Stroke etiology influences both prognosis and outcome[13].

Survivability of acute stroke patients reduces post-stroke and are highly susceptible to recurrent stroke, therefore needing improved patient care. Globally and locally, there were few studies that have reported on the survivability of post-stroke patients, however, it differs in terms of the conduct of the study, the study subjects, and the design [14–17]. Additionally, there are numerous studies that have reported on stroke-related mortalities in their region [18]. A study on the short-term and long‐term survival was conducted in Malaysia among first-ever ischemic and hemorrhagic stroke patients to determine the 28‐day, 1‐year, and 5‐year survival. It reported that the survival probabilities were 78.0% (28‐day), 74.2% (1-year) and 70.9% (5-year) [19].

Age, gender, type and severity of stroke, history of stroke, diabetes, and heart disease have been identified as the risk factors that influenced post-stroke mortality [2]. Data from Malaysia report a continuously increasing prevalence of vascular risk, especially diabetes, hyperlipidemia and obesity. In addition, data on government hospital admissions from 2008 to 2016 showed a significant increase in stroke incidence in those under 65 years of age in both sexes [20].

Thrombolytics, antiplatelets, and anticoagulants, which are commonly used in stroke treatment, have a high risk of causing serious hemorrhagic manifestations. Routine monitoring is strongly advisable for stroke patients owing to the existence of multiple risk factors such as polypharmacy, prolonged therapy, medication errors, and comorbidities [21].

As indicated by the Health Director-General of Malaysia, there is a dire need for neurologists to cater to the high number of stroke patients in the country. Malaysia has 107 neurology specialists nationwide with only 50 specialists and trainees in the healthcare sector [22]. In 2019, the neurologist-to-patient ratio was 1:323,000 [23]. He also added that it will take a long time to attain a sufficient number of neurology specialists and that specialists from other departments could also lend a hand in providing early assistance for stroke patients [22]. Moreover, the actual number of stroke units in Malaysia is not known [17].

The primary objective of this research is to access the survivability of stroke patients admitted to the primary stroke center in Penang, Malaysia. The secondary objective of this study is to describe, the most common drugs prescribed to these patients. This is the first study in Malaysia to report on research on the survivability of patients’ post-stroke survival, using data from the local stroke registry and medical records that further validates the findings. It is hoped that the findings of this study will encourage more Malaysian non-stroke hospitals to increase their efforts in treating stroke patients. Furthermore, it hoped to assist health professionals in making decisions about the prognosis and management of this vulnerable group, which will lead to improved clinical care for stroke patients.

## Methods

### Study setting

This study was conducted at Hospital Seberang Jaya (HSJ), Malaysia. This government health facility serves as the primary stroke center in Penang, Peninsular Malaysia. HSJ’s six-bed Acute Stroke Unit (ASU) was dedicated to the care of post-stroke patients.

### Study design and data collection

This 5-year study enrolled patients hospitalized for stroke from 2016 to 2020. Hospitalized stroke patients were initially identified from a stroke database maintained by Clinical Research Center Hospital Seberang Jaya (CRCHSJ). Subsequently, their medical records were then accessed for data collection, which included demographic information, comorbid conditions, and medications prescribed during admission.

Data was extracted from medical records by one researcher and validated by another. The patient must be admitted for stroke and admitted for more than 24 h to meet the inclusion criteria. Patients whose admission was not due to a stroke, as well as those with incomplete or questionable medical records on the reason for admission, will be excluded from this study.

### Statistical analysis

For the purpose of descriptive analysis, baseline characteristics of patients with stroke were analyzed using either Pearson’s chi-square test for categorical variables and t-test or Mann-Whitney test, depending on the skewness of data, for continuously distributed variables.

### Survival analysis and Cox regression

The Kaplan-Meier survival data estimates have become a standard of reporting the varying survival times of the subjects that is based on the time-to-event [24]. In this study the time noted was the duration of hospitalization and the event was either alive or dead at the time of discharge. The survivability of the post-stroke patients was reported based on the percentage of cumulative survival corresponding to the days of hospitalization. Subsequently, the inter variable comparison between the survived and the those who didn’t survive was done using the log rank test, and the significance value was reported. The cumulative survival was reported graphically using the Kaplan-Meier estimates, plotting the log-minus survival function over time.

The Cox regression analysis was used to estimate of the relative risk of outcome in relation to time, which was reported as hazard ratio [25]. The length of hospitalization was used as the time, with the outcome being either survival or death. The advantage of using Cox regression model is the ability to censor patients who fail to reach the study end-point [25]. In this case, patients who survived stroke were censored. Each risk factor was subjected to univariate Cox regression analysis, followed by multiple Cox regression.

All analysis was performed using SPSS (version 22; SPSS Inc., Chicago, IL). Two-sided p-values of less than 0.05 were considered statistically significant.

## Results

### Univariate analysis

**Fig. 1:**
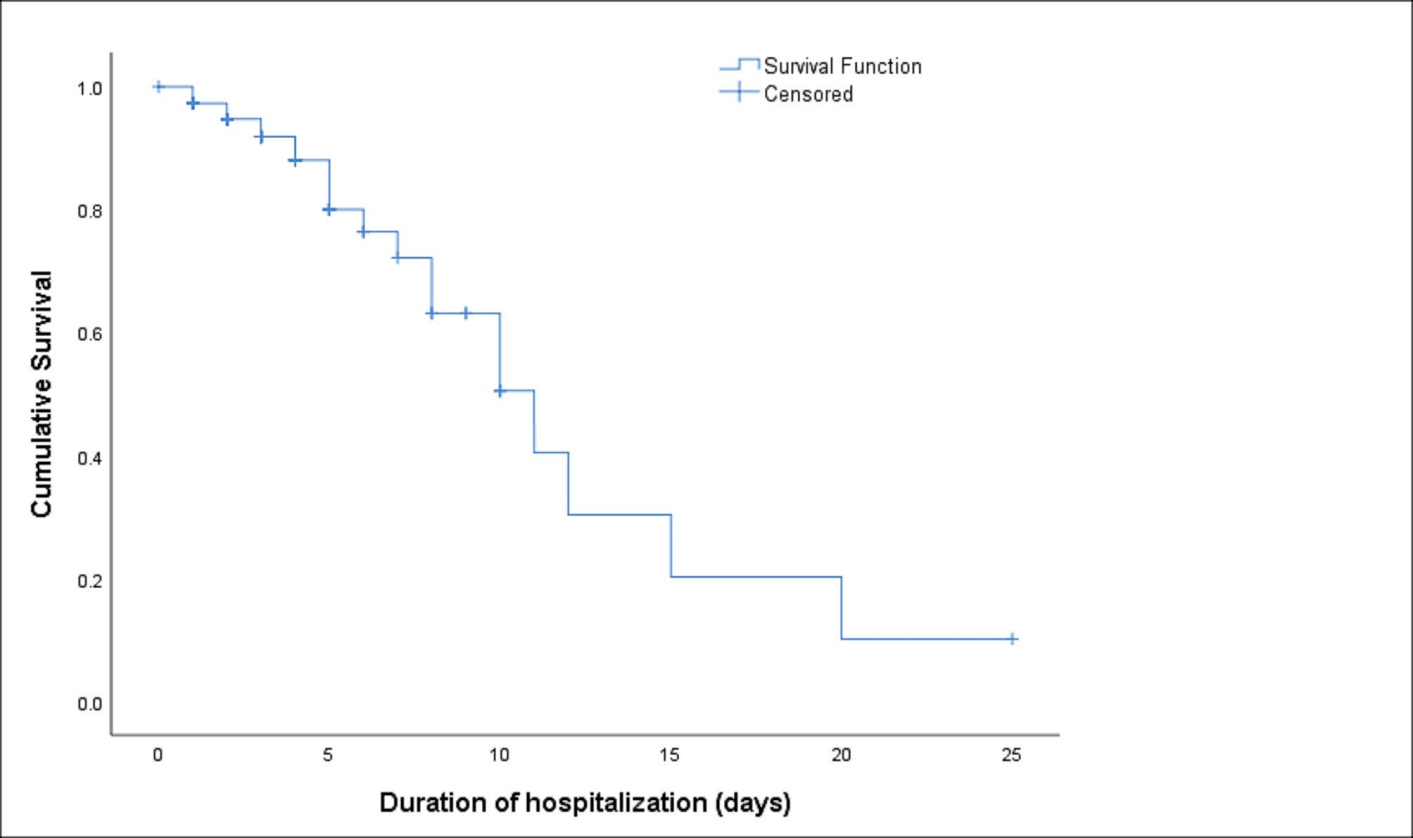
Overall survivability for patients who were admitted for stroke at General Hospital Seberang Jaya

One thousand and nine hundred and ninety-three stroke patients were identified using the stroke registry database maintained by CRCHJ, Penang Malaysia from year 2016 till 2020, out of which only 185 medical records fulfilled the requirements of the inclusion and exclusion criteria. Of the 185 eligible patients, 160 survived and 25 did not survived post-stroke for this study duration. (Fig. 1: **Data collection flow chart**) (Danial M, Mohdradzi NSI, Khan AH, Ch’ng ASH, Looi Irene. Risk factors of patients admitted for stroke in a primary referral center for general healthcare facility of mainland Penang, Malaysia: A retrospective 5-year study, submitted).

### Cumulative survivability analysis using Kaplan-Meier

**Fig. 2:**
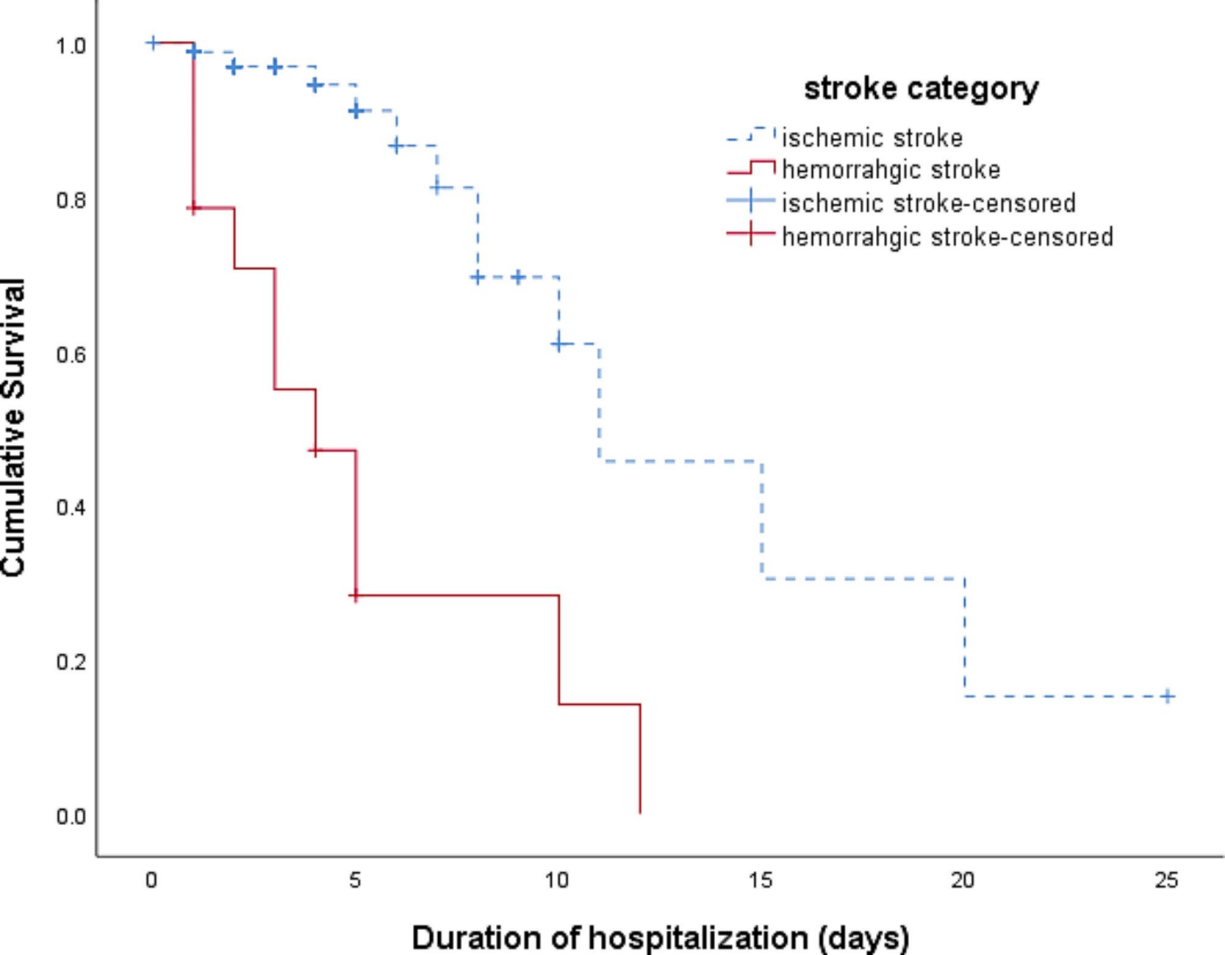
Survivability for patients who were admitted for stroke based on stroke category at General Hospital Seberang Jaya

**Fig. 3:**
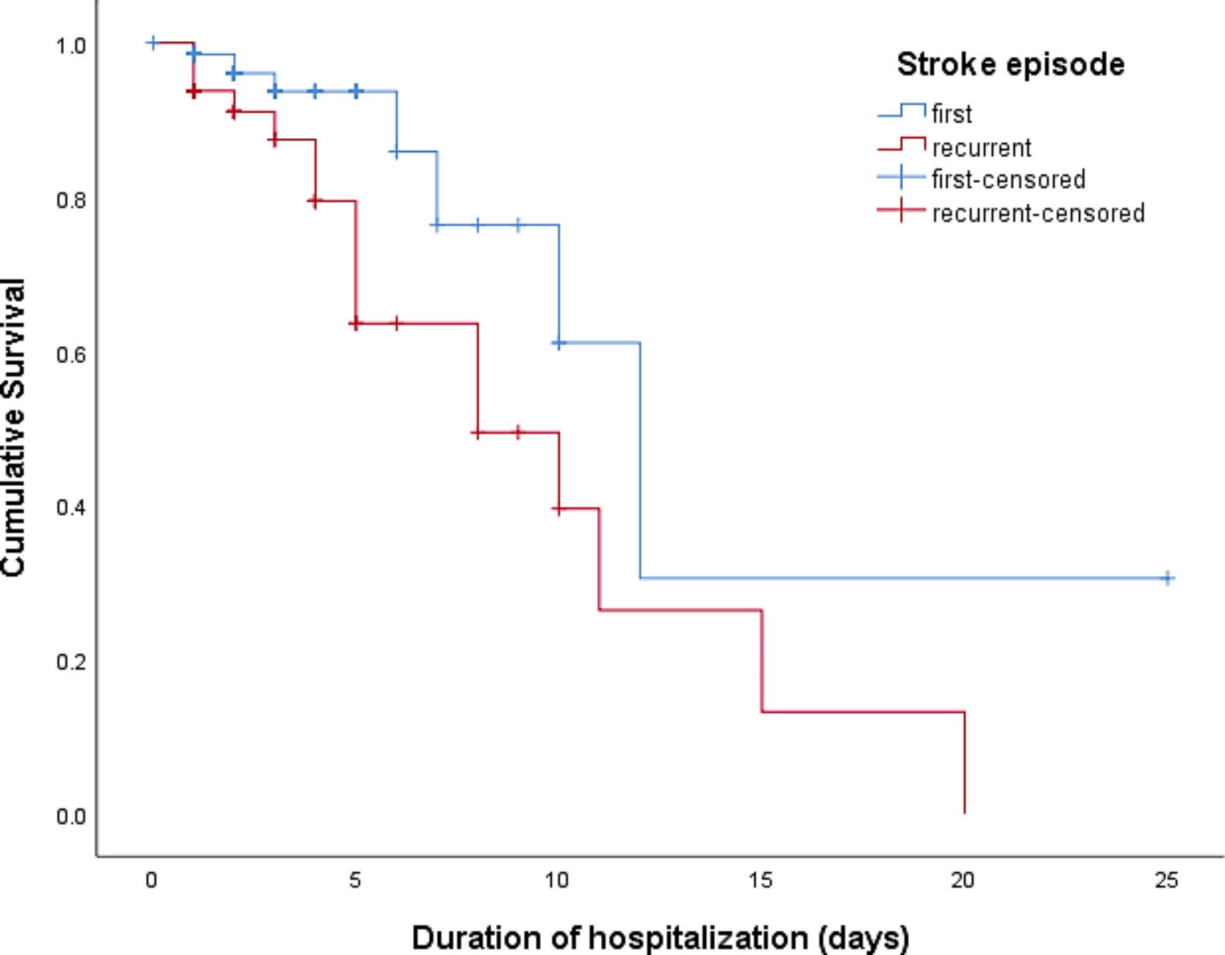
Survivability for patients who were admitted for stroke based on stroke episode at General Hospital Seberang Jaya

The Kaplan-Meier overall survivability analysis performed indicated 50.5% survival for the duration of 10 days (Fig. 1). Ten-day survival rates for patients with ischemic stroke and hemorrhagic stroke were 60.9% and 14.1% (p < 0.001) (Fig. 2), and for stroke episodes were 61.1% (first) and 39.6% (recurrent) (p < 0.001) (Fig. 3). Additionally, the survival rates observed for patients prescribed and not prescribed with anti-platelets were 46.2% (prescribed) and 41.5% (not prescribed) (p = 0.014); statins 68.7% (prescribed) and 28.1% (not prescribed) (p < 0.001); anti-hypertensive 65.4% (prescribed) and 45.9% (not prescribed) (p = 0.046); and anti-infectives 42.5% (prescribed) and 59.6% (not prescribed) (p = 0.005) respectively. However, no survival differences were observed in categories of age, gender, ethnicity, smoking status, number of comorbidities, number of medications, proton pump inhibitor prescription and anti-diabetic drugs prescription. Ten days survival rates of for the above categories were age groups ≤ 61 years and ≥ 62 years were 51.6% and 52.2%; gender were 46.6% male and 32.8% female; ethnic groups were 65.2% Malay, 0% Chinese, 0% Indian and 43.0% others; smoking status were 48.0% non-smokers and 68.8% smokers; number of comorbidities were 53.5% (2 or less comorbidities) and 26.5% (3 or more comorbidities); with the uptake of 10 or less and 11 or more medications were 0% and 60.8%; proton pump inhibitors 41.1% (prescribed) and 40.5% (not prescribed); and anti-diabetic medication 41.1% (prescribed) and 35.7% (not prescribed) respectively (Table 1).

**Table 1:** Survivability of post-stroke patients based on risk factors hospitalized at Hospital Seberang Jaya using Kaplan-Meier analysis (n = 185)

Variables		Number of	Number of	Survivability (%)					*p*-value^a^
		Patients, n	Events,	2 days	4 days	6 days	8 days	10 days	
			n						
**Overall Survivability**		**185**	**25**	**94.7**	**88.0**	**76.4**	**63.1**	**50.5**	
**Age category**									0.905
	≤ 61 years	95	13	94.4	87.7	75.6	61.9	51.6	
	≥ 62 years	90	12	96.7	95.0	88.3	69.6	52.2	
**Gender**									0.895
	Male	92	15	94.0	86.1	69.1	62.2	46.6	
	Female	68	13	96.4	91.8	78.7	65.6	32.8	
**Ethnicity**									0.750
	Malay	87	9	91.8	87.0	87.0	65.2	65.2	
	Chinese	64	10	89.9	85.1	61.3	49.0	0.0	
	Indian	25	3	75.0	75.0	75.0	50.0	0.0	
	Others	9	3	87.5	87.5	87.5	87.5	43.0	
**Smoking status**									0.124
	No	137	23	93.0	86.9	76.8	61.7	48.0	
	Yes	48	2	91.7	91.7	68.8	68.8	68.8	
**Stroke category**									< 0.001*
	Ischemic stroke	171	14	96.9	94.6	86.6	69.6	60.9	
	Hemorrhagic stroke	14	11	70.7	47.1	28.3	28.3	14.1	
**Stroke episode**									< 0.001*
	First	137	9	96.0	93.7	85.9	76.3	61.1	
	Recurrent	48	16	91.1	79.5	63.6	49.5	39.6	
**Number of comorbidities**									0.136
	≤ 2comorbidities	107	10	96.5	93.7	93.7	75.0	53.5	
	≥ 3comorbidities	78	15	92.5	82.7	61.9	53.1	26.5	
**Number of medications**									0.118
	≤ 10medications	103	10	94.2	83.7	73.2	73.2	0.0	
	≥ 11medications	82	15	95.6	90.6	79.0	68.5	60.8	
**Anti-platelets**									0.014*
	No	29	17	82.6	67.6	53.8	48.4	41.5	
	Yes	156	8	99.4	91.4	83.1	69.3	46.2	
**Statins**									< 0.001*
	No	33	18	84.0	67.4	49.1	35.1	28.1	
	Yes	152	7	97.1	92.5	85.9	85.9	68.7	
**Proton pump inhibitors**									0.863
	No	49	5	93.9	82.1	82.1	41.1	41.1	
	Yes	136	20	94.9	85.8	74.8	65.1	40.5	
**Anti-diabetic**									0.266
	No	112	18	93.6	85.6	70.3	57.5	41.1	
	Yes	73	7	96.4	91.8	71.4	71.4	35.7	
**Anti-hypertensive**									0.046*
	No	127	20	94.2	83.6	71.3	55.1	45.9	
	Yes	58	5	95.9	87.2	87.2	87.2	65.4	
**Anti-infective**									0.005*
	No	150	5	97.5	89.3	89.3	59.6	59.6	
	Yes	35	20	85.7	73.0	61.9	53.1	42.5	

Note: ^a^ survival analysis using Kaplan-Meier

### Mortality risk analysis using Cox regression

The Cox regression analysis revealed that factors such as stroke category, number of comorbidities, no prescriptions for statins and anti-diabetic medications, and prescriptions for anti-infectives all had a significant impact on post-stroke mortality (p < 0.050). Other factors were not significantly linked to mortality after stroke among the subjects (Table 2). Stroke patients whom had hemorrhagic stroke had 10.61 higher risk of mortality compared to those with ischemic stroke (p = 0.004). Patients who were with 3 or more comorbidities had 6.60 higher risk of mortality compared with patients with 2 or less comorbidities (p = 0.020). Patients prescribed with statins and anti-diabetic had lower mortality risk of 0.25 (p = 0.050) as compared with patients who were not prescribed with neither medication. Patients prescribed anti-infectives, on the other hand, had a higher risk of mortality when compared to patients who did not receive anti-infectives (HR: 13.10, p = 0.019).

**Table 2:** Factors associated with mortality using Cox regression analysis for patients who were admitted for stroke at General Hospital Seberang Jaya (n = 185)

Variables		Censored		Events		Simple. HR	(95% CI)	*p*-value^a^	Adj. HR	(95% CI)	*p*-value^b^
		n	(%)	n	(%)						
**Age category**								0.907			0.240
	≤ 61 years	95	87.9	13	12.1	1.00	(ref.)		1.00	(ref.)	
	≥ 62 years	90	88.2	12	11.8	1.05	(0.47, 2.32)		0.44	(0.11, 1.72)	
**Gender**								0.896			0.069
	Male	92	86.0	15	14.0	1.00	(ref.)		1.00	(ref.)	
	Female	68	82.9	13	17.1	0.95	(0.41, 2.20)		0.26	(0.06, 1.11)	
**Ethnicity**								0.765			0.257
	Malay	87	90.6	9	9.4	1.00	(ref.)		1.00	(ref.)	
	Chinese	64	86.5	10	13.5	1.67	(0.64, 4.37)		0.65	(0.19, 2.23)	
	Indian	25	89.3	3	10.7	1.17	(0.30, 4.52)		0.86	(0.13, 5.63)	
	Others	9	75.0	3	25.0	1.27	(0.31, 5.13)		0.08	(0.01, 0.95)	
**Smoking status**								0.147			0.124
	No	137	85.6	23	14.4	1.00	(ref.)		1.00	(ref.)	
	Yes	48	96.0	2	4.0	0.34	(0.08, 1.46)		0.19	(0.02, 1.57)	
**Stroke category**								< 0.001*			0.004*
	Ischemic stroke	171	92.4	14	7.6	1.00	(ref.)		1.00	(ref.)	
	Hemorrhagic stroke	14	56.0	11	44.0	6.60	(2.86, 15.21)		10.61	(2.1, 53.68)	
**Stroke episode**								0.029*			0.245
	First	137	93.8	9	6.2	1.00	(ref.)		1.00	(ref.)	
	Recurrent	48	75.0	16	25.0	2.54	(1.10,5.89)		1.99	(0.62, 6.32)	
**Number of comorbidities**								0.148			0.020*
	≤ 2comorbidities	107	91.5	10	8.5	1.00	(ref.)		1.00	(ref.)	
	≥ 3comorbidities	78	83.9	15	16.1	1.18	(0.81, 4.09)		6.60	(1.34, 32.44)	
**Number of medications**								0.129			0.054
	≤ 10medications	103	91.2	10	8.8	1.00	(ref.)		1.00	(ref.)	
	≥ 11medications	82	84.5	15	15.5	0.49	(0.19, 1.23)		0.19	(0.04, 1.03)	
**Anti-platelets**								0.019*			0.068
	No	29	63.0	17	37.0	1.00	(ref.)		1.00	(ref.)	
	Yes	156	95.1	8	4.9	0.32	(0.12,0.83)		4.87	(0.89, 26.58)	
**Statins**								< 0.001*			0.050*
	No	33	64.7	18	35.3	1.00	(ref.)		1.00	(ref.)	
	Yes	152	95.6	7	4.4	0.18	(0.07,0.44)		0.25	(0.06, 1.00)	
**Proton pump inhibitors**								0.866			0.440
	No	49	90.7	5	9.3	1.00	(ref.)		1.00	(ref.)	
	Yes	136	87.2	20	12.8	0.92	(0.33,2.54)		0.50	(0.09, 2.90)	
**Anti-diabetic**								0.278			0.006*
	No	112	86.2	18	13.8	1.00	(ref.)		1.00	(ref.)	
	Yes	73	91.3	7	8.8	0.62	(0.26,1.48)		0.13	(0.03, 0.55)	
**Anti-hypertensive**								0.060			0.379
	No	127	86.4	20	13.6	1.00	(ref.)		1.00	(ref.)	
	Yes	58	92.1	5	7.9	0.35	(0.12,1.04)		0.52	(0.12, 2.22)	
**Anti-infective**											0.019*
	No	150	96.8	5	3.2	1.00	(ref.)	0.009*	1.00	(ref.)	
	Yes	35	63.6	20	36.4	4.38	(1.45,13.25)		13.10	(1.54, 111.41)	

Note: ^a^Univariate Cox Regression; ^b^Multivariate Cox Regression

Simple HR (95% CI) = Simple hazard ratio (95% confidence interval) ; Adj. HR (95% CI) = Adjusted hazard ratio (95% confidence interval)

### Medication prescription for patients admitted for stroke

The major drug classes prescribed for patients who were admitted for stroke at HSJ were anti-platelets (n = 156 [86.7%]) which comprises aspirin (n = 130 [48.3%]), clopidogrel (n = 91 [33.8%]) and glyprin (n = 25 [9.3%]) ; statins (n = 152 [84.4%]) which comprises of simvastatin (n = 99 [59.3%]) and atorvastatin (n = 68 [40.7%]); proton pump inhibitors (n = 136 [75.6%]) which comprises of pantoprazole (n = 134 [96.4%]) and omeprazole (n = 5 [3.6%]); anti-diabetic (n = 73 [40.5%]) which comprises of metformin (n = 55 [42.3%]), actrapid (n = 28 [21.5%]) and gliclazide (n = 24 [18.5%]); anti-hypertensive (n = 65 [36.1%]) which comprises of perindopril (n = 61 [88.4%]), losartan (n = 3 [4.3%]) and telmisartan (n = 2 [2.9%]); and anti-infective (n = 35 [19.4%]) which comprises of amoxicillin (n = 23 [36.5%]), ampicillin (n = 9 [14.2%]), carbapenem (n = 6 [9.5%]) and ceftazidime (n = 4 [6.3%]) (Table 3).

**Table 3:** Major drug classes and drugs prescribed for patients who were admitted for stroke at General Hospital Seberang Jaya (n = 185)

Major Drug Class	Patients, n (%)	Drug name	n (%)
Anti-platelets	156 (86.7%)	Aspirin	130 (48.3%)
		Clopidogrel	91 (33.8%)
		Glyprin	25 (9.3%)
		Others	23 (8.6%)
Statins	152 (84.4%)	Simvastatin	99 (59.3%)
		Atorvastatin	68 (40.7%)
Proton pump inhibitors	136 (75.6%)	Pantoprazole	134 (96.4%)
		Omeprazole	5 (3.6%)
Anti-diabetic	73 (40.5%)	Metformin	55 (42.3%)
		Actrapid	28 (21.5%)
		Gliclazide	24 (18.5%)
		Others	23 (17.7&)
Anti-hypertensive	65 (36.1%)	Perindopril	61 (88.4%)
		Losartan	3 (4.3%)
		Telmisartan	2 (2.9%)
		Others	3 (4.3%)
Anti-infective	35 (19.4%)	Amoxicillin	23 (36.5%)
		Ampicillin	9 (14.2%)
		Carbapenem	6 (9.5%)
		Ceftazidime	4 (6.3%)
		Others	21 (33.3%)

## Discussion

From our study findings, the survivability of patients who were admitted for stroke were significantly dependent on the type of stroke category with those patients that had a hemorrhagic stroke had higher risk of mortality (HR: 10.61) compared with those that had an ischemic stroke. Therefore, it is evident that hemorrhagic have a higher impact on the survivability post stroke. The findings were consistent with previous reports in which the mortality rates of patients that had hemorrhagic stroke were 2-3-fold higher compared with patients that had ischemic stroke [19, 26–28]. Comparatively, hemorrhagic stroke has lower prognosis compared to ischemic stroke and is often fatal [29].

Ten days survival rates of patients with ischemic stroke and hemorrhagic stroke were 60.9% and 14.1% respectively as reported in this study which were lower compared to other post stroke survivability studies done [30–32]. These were attributed to the higher level of knowledge on stroke among the general public and rapid response of seeking treatment on the onset of stroke [33]. Furthermore, the higher survivability rate was attributed to these patients being cared for in a stroke unit by a multidisciplinary stroke team, which resulted in a higher 1-month survival probability from ischemic stroke (87.2%) and hemorrhagic stroke (82.2%) [34]. Malaysia Stroke Council initiated efforts to instill stroke knowledge in the public, introducing the Fast Heroes campaign, which allows for the rapid identification of stroke symptoms [35].

Stroke episodes were important risk factors in our study as the patients with recurrent stroke episode has higher mortality compared with patients that had experienced their first stroke episode. Recurrent ischemic stroke has been linked to increased mortality and functional dependence, as it was a strong independent factor doubling mortality estimates ranging from a 2-fold to a 17-fold increase [16, 36]. A study conducted by Jørgensen et al. (1997) reported that patients with contralateral recurrent stroke have more severe functional disability than those with ipsilateral recurrence, which reduces the brain’s ability to compensate [37].

From our study findings anti-platelets, statins and proton pump inhibitors were the primary major drug classes that were used in treatment of the stroke patients. Rapid treatment with an antiplatelet drug, such as aspirin, prevents new clots from forming and improves recovery after stroke, Antiplatelet therapy, initiated within 48 h of stroke onset, significantly reduced mortality and toxicity, reduced the risk of recurrent stroke without a major risk of early hemorrhagic complications, and improved long-term outcomes [38], therefore improving the patients survivability post-stroke. The statins primarily used in our study were simvastatin and atorvastatin. Statins have neuroprotective effects that are significantly associated with improved functional outcomes at hospital discharge [39]. Protective effects may occur through mechanisms other than lipid-lowering, such as alteration of endothelial function, anti-inflammatory effects, enhanced plaque stability, and reduced thrombus formation [40]. Stroke patients are at high risk of developing pneumonia, which is the leading cause of death after stroke. Proton pump inhibitors and H2 receptor blockers are anti-ulcer drugs that may predispose to the development of pneumonia by suppressing gastric acid through their bactericidal action [41, 42].

Additionally, the findings from our study shows that stroke patients who were prescribed with anti-infectives had a higher rate of mortality which could be due to stroke-induced immunosuppression, a systemic anti-inflammatory which increases the susceptibility to infection [43]. Several studies found that infection was linked to poor functional outcome and mortality, while others reported that infections were just a marker of stroke severity with no impact on clinical outcome [43, 44].

Data from 14 Malaysian public hospitals revealed suboptimal use of antihypertensive drugs and anticoagulants among ischemic stroke patients [17]. To address these deficiencies, internal audits, close supervision, and patient follow-up can all be improved. At the same time, education is an important means of increasing health care workers’ knowledge and skills whish are hoped to overcome imbalances caused by a lack of specialists and neurologists, as well as a misallocation of allied healthcare personnel [23, 45].

### Study significance and limitation

With the incorporation of evidence-based data, this study contributes to local data for comparison and improves the implementation of regularly prescribed stroke medication. Unlike previous studies that relied solely on registry data, the data for this study was obtained from the local stroke registry and medical records, which validates the findings even further. Furthermore, the study was conducted in a primary stroke center in Penang, Malaysia, thus increasing the reliability of the study findings. Lastly, it is hoped that the findings of this study will lead to more non-neurological facilities becoming more knowledgeable in treating stroke patients. The fact that this study was conducted in hospitals means that results may differ from hospital to hospital due to differences in community characteristics and hospital specialties. Also, the survivability of these patients was only assessed during hospitalization and not after discharge.

## Conclusion

The study’s findings are intended to encourage more non-stroke hospitals in Malaysia to increase their efforts in treating stroke patients, as prompt treatment can help to reduce the severity of the stroke. More efforts should be made to educate the community about stroke prevention. Clinician involvement, intensive resources, and regular monitoring are required to improve stroke care. Lastly, clinician should be aware of the potential impact of drug-drug interactions and frequent complication of stroke patients.

## Data Availability

The datasets generated and analysed during the current study are not publicly available due to data confidentiality policy as dictated in the study approval letter by the Medical Research & Ethics Committee (MREC), Ministry of Health Malaysia (MOH) (Ref no: NIH.800-4/4/1 Jld.116(06)) but are available from the corresponding author on reasonable request.
